# Basal endothelial glycocalyx’s response to shear stress: a review of structure, function, and clinical implications

**DOI:** 10.3389/fcell.2024.1371769

**Published:** 2024-03-18

**Authors:** Zoe Vittum, Samantha Cocchiaro, Solomon A. Mensah

**Affiliations:** ^1^ Biomedical Engineering Department, Worcester Polytechnic Institute, Worcester, MA, United States; ^2^ Mechanical Engineering Department, Worcester Polytechnic Institute, Worcester, MA, United States

**Keywords:** endothelial glycocalyx, heparan sulfate, syndecan 4, syndecan 1, basal glycocalyx, mechanotransduction

## Abstract

The endothelial glycocalyx encompasses the entire endothelial cell, transducing extracellular signals and regulating vascular permeability and barrier functions. The apical glycocalyx, which forms the lumen of the vessel, and the basal glycocalyx, at the smooth muscle cell interface, are often investigated separately as they are exposed to vastly different stimuli. The apical glycocalyx directly senses fluid shear forces transmitting them intracellularly through connection to the cytoskeleton of the endothelial cell. The basal glycocalyx has demonstrated sensitivity to shear due to blood flow transmitted through the cytoskeleton, promoting alternate signaling processes. In this review, we discuss current literature on the basal glycocalyx’s response to shear stress in the context of mechanotransduction and remodeling. The possible implications of basal glycocalyx degradation in pathologies are also explored. Finally, this review seeks to highlight how addressing the gaps discussed would improve our wholistic understanding of the endothelial glycocalyx and its role in maintaining vascular homeostasis.

## Introduction

**FIGURE 1:**
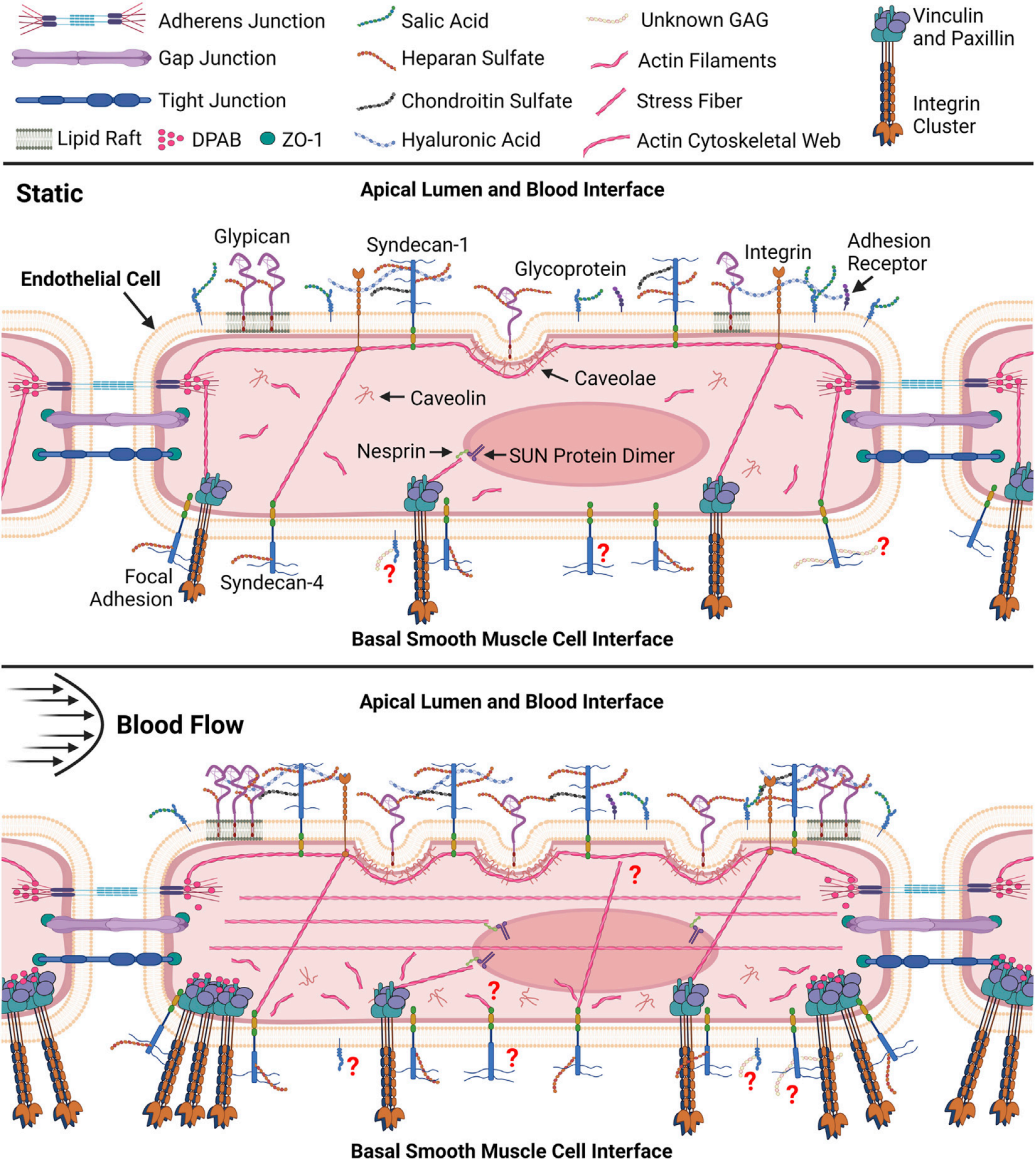
Pictorial representation (not to scale) of resting endothelial cell and comprehensive endothelial cell remodeling response to fluid shear. Within the static endothelial cell syndecan-1 and integrins can be seen connected to the apical cytoskeletal web which associate with dense peripheral actin bands (DPAB) surrounding tight junctions (Thi et al., 2004). Actin bands can also be seen in both cells interfacing with Nesprin proteins that further attach to SUN dimer proteins embedded in the nuclear envelope allowing for transmission of tension in the cytoskeleton to the nucleus (Kaunas et al., 2005; Rajgor and Shanahan, 2013). DPABs are also shown to associate with stress fibers connected to focal adhesions in the basal cell in the static environment. Under shear these stress fibers are shown to associate with DPABs to support new focal adhesions at the smooth muscle cell (SMC) interface while stress fibers form throughout the apical cell (Thi et al., 2004). Li and colleagues further discuss the details of the vascular endothelial cell and smooth muscle cell interface and their interactions (Li et al., 2018). Disorganized actin filaments are shown throughout the basal cell after shear exposure (Zeng and Tarbell, 2014). Stress fibers are also shown between apical integrins and basal syndecan-4 and focal adhesions in both cells (Thi et al., 2004). Application of shear is shown to promote new caveolae formation which are anchored to the cytoskeletal web through caveolin in the apical cell (Zeng and Tarbell, 2014). Caveolin house associated glypican molecules however glypican can also be bound to mobile lipid rafts shown to increase in the peripheral apical cell after shear exposure (Zeng and Tarbell, 2014). The presence of syndecan-1 is also shown to increase with shear exposure. Question marks indicate gaps in our knowledge relating to the basal glycocalyx. For example, question marks surrounding unknown basal glycosaminoglycans (GAGs) indicate our lack of knowledge surrounding GAGs in the basal glycocalyx (Longley et al., 1999; Thi et al., 2004; Weinbaum et al., 2007; Zeng and Tarbell, 2014; Nikmanesh et al., 2019; Mensah et al., 2021). Created with BioRender.com.

The endothelial glycocalyx is a transmembrane polysaccharide grass-like structure that protrudes from endothelial cells and serves to transduce extracellular mechanical stimuli for intra- and extra-cellular signaling ultimately influencing vascular permeability, tone, inflammation, and signals (Foote et al., 2022; Jin et al., 2021; Reitsma et al., 2007). The apical side of endothelial cells form the lumen of the vessel and are exposed to blood flow while the basal endothelial cell interacts with the smooth muscle and other basement membrane proteins. The original mention of the basal glycocalyx was documented in a 1903 paper, yet it was unpublished until 1974 (Lushbaught and Miller, 1974). This research specifically investigated the basal glycocalyx of entamoeba histolytica (Lushbaught and Miller, 1974). Subsequently, in 1981, Ausprunk et al. showcased the existence of a basal glycocalyx on endothelial cells in Rabbit Corneas (Ausprunk et al., 1981). Nevertheless, since that time, there has been a lack of substantial research conducted on the basal endothelial glycocalyx. Most research efforts remain centered on studying the apical glycocalyx. The apical endothelial glycocalyx is mainly composed of negatively charged glycoproteins and proteoglycans (Bai and Wang, 2014; Curry and Adamson, 2012; Foote et al., 2022; Reitsma et al., 2007). As seen in Figure 1, Glycoproteins are embedded in the lipid bilayer with carbohydrate chains attached to them which are typically short and capped with neuraminic acid sugar residues (Reitsma et al., 2007; Foote et al., 2022). Most of the glycoproteins on the endothelial cell surface are cell adhesion molecules, integrin families, or selectin cellular receptors (Reitsma et al., 2007; Foote et al., 2022). Other cell adhesion molecules which interact with the apical endothelial glycocalyx include intercellular adhesion molecules, platelet endothelial cell adhesion molecules, and vascular cell adhesion molecules (Foote et al., 2022). Proteoglycans on the other hand are larger glycoproteins that have glycosaminoglycan (GAG) chains attached, densely surrounding other smaller glycoproteins (Reitsma et al., 2007; Foote et al., 2022; Merry et al., 2022) There are many proteoglycans that make up the apical glycocalyx such as biglycan, decorins, mimecans and perlicans, but the two most important families of proteoglycans are syndecans and glypicans (Reitsma et al., 2007; Jin et al., 2021; Foote et al., 2022). Syndecans attach to the endothelial cell via membrane-spanning domains (Reitsma et al., 2007; Jin et al., 2021; Foote et al., 2022). There are four syndecan members (syndecan −1 to −4). Syndecan-1 is a crucial component of the apical glycocalyx, holding a pivotal role in modulating cell behavior, inflammation, and transmitting external mechanical forces to the entire cell through its connections with specific proteins in the cortical cytoskeleton. It possesses five sites where GAGS can attach (Figure 1), contributing significantly to its function (Reitsma et al., 2007; Foote et al., 2022; Merry et al., 2022). Glypicans attach to the endothelial cell membrane via glycosylphosphatidylinositol molecules within the outer plasmalemma (Foote et al., 2022). There are six glypican members (glypican −1 to −6) but glypican-1 is mostly found in the apical endothelial glycocalyx functioning to mediate shear stress mechanotransduction and other signaling pathways (Figure 1) (Reitsma et al., 2007; Jin et al., 2021; Foote et al., 2022). CD44 is a glycosylated membrane receptor that plays a large role in signal transduction, cytoskeleton remodeling, and cell adhesion, in a healthy glycocalyx CD44 is covered by a sea of GAG chains (Azevedo et al., 2018; Mensah et al., 2021). While both proteoglycans and glycoproteins contribute to providing structural support for the glycocalyx, proteoglycans are regarded as the primary foundation of the endothelial glycocalyx. This status stems from their close association with GAGs, which significantly influences their structural and functional roles within the glycocalyx (Foote et al., 2022). GAGs are long chains of repeating disaccharide units, the chains are highly polar and have sulfonation patterns which modulate specific protein attachments, influencing the glycocalyx’s electrical charge and the glycocalyx functions (Foote et al., 2022). The main GAG chains include hyaluronan, chondroitin sulfate, various moieties of sialic acids, dermatan sulfate, and heparan sulfate (Figure 1) (Reitsma et al., 2007; Jin et al., 2021; Foote et al., 2022). GAG chains attach to the glycoproteins and proteoglycans via specific attachment sites on their extracellular domains (Eriksson and Spillmann, 2012; Bhattacharya et al., 2017).

It is evident that while extensive research has elucidated the components and responses of the apical glycocalyx to shear stress, the same depth of understanding is lacking for the basal endothelial glycocalyx. Researchers acknowledge the distinct component composition of the basal glycocalyx, implying potentially different intra- and extra-cellular responses (Zeng and Tarbell, 2014). An example lies in the types of syndecans present on both the apical and basal glycocalyx: Syndecan-1, primarily apical, contrasts with Syndecan-4, predominantly basal and colocalized with adhesion molecules, integrins, and cytoskeletal elements to form focal adhesions (Saoncella et al., 1999; Hahn et al., 2011; Foote et al., 2022; Merry et al., 2022). Table 1 highlights other known differences in the apical and basal glycocalyx structure, composition, and shear response. The connection between the actin cortical web and the glycocalyx—both apical and basal—is recognized (Thi et al., 2004). The “bumper-car model,” introduced by Thi et al., in 2014, proposes that shear stress applied to the apical endothelial glycocalyx transmits signals from apical integrins through the actin cortical web to vinculin, paxillin, and syndecan-4, a basal glycocalyx component (Figure 1) (Thi et al., 2004). Consequently, shear stress affecting the apical glycocalyx also influences the basal membrane, impacting pathways like YAP/TAZ and Rho (Elfenbein and Simons, 2013). The impact of shear stress on the apical glycocalyx contributes to both glycocalyx degradation and synthesis (Liu et al., 2016; Harding et al., 2018; Foote et al., 2022). Understanding how shear stress affects the basal glycocalyx could significantly aid in disease research and treatment. Atherosclerosis, cancer, and sepsis which are known to influence the apical glycocalyx, may also affect the basal glycocalyx. This review aims to delve into the current knowledge gaps regarding the basal glycocalyx in comparison to the apical glycocalyx in endothelial cell mechanotransduction and remodeling. Furthermore, it seeks to highlight how addressing these gaps could provide a wholistic view of the endothelial glycocalyx and its potential impact on disease onset and progression.

**TABLE 1:** Summary table of known apical and basal glycocalyx structure, function, and response to shear. The known overall functions and compositional differences in core proteins and GAGs of the apical and basal glycocalyx are noted. Green shading indicates the glycocalyx component is known while red shading indicates the core protein or GAG is not known to be present in the apical or basal glycocalyx. Mechanotransduction of shear forces is expanded upon to highlight differences in flow stimulation, cytoskeletal remodeling, and signal transduction pathways (eNOS- nitric oxide synthase, CD59-complimentary-inhibitory proteins clusterin, SOD-superoxide dismutase, HO-1-heme oxygenase-1, NQO1-NAD(P)H quinoneo oxidoreductase 1, PRX-peroxiredoxins, MKP-1-mitogen-activated protein kinase phosphate, Ang-2-angiopoitein-2, SREBP-sterol regulatory element binding protein, TLR-2- Tall-like receptor, *etc.*) (Davies, 1995; Tarbell and Pahakis, 2006).

	Apical	Basal
**Overall Function**	- Regulate vascular permeability and barrier functions Curry and Adamson (2012)	- Intercellular interactions between endothelial cells and smooth muscle cells (Li et al. (2018)
	- Intercellular interactions between multiple endothelial, immune, and cancer cells Mensah et al. (2020), Mensah et al. (2021)	- Mechanotransduction Elfenbein and Simons (2013)
	- Interprotein interactions between extracellular receptors (VCAM-1, ICAM-1, E-selectin ect.) Qu et al. (2021)	
	- Attenuation of leukocytes and platelet adhesion Dogné and Flamion (2020), Qu et al. (2021)	
	- Mechnaotransduction and others as reviewed by Fu & Tarbell Fu and Tarbell (2013), Zeng et al. (2018)	

**Known Core Proteins** Reitsma et al. (2007), Foote et al. (2022)	1–4	**Syndecan**	4 Chronopoulos et al. (2020)
	1–6	**Glypican**	
		**Perlecan**	
		**Versican**	
		**Decorin, Biglycan, *etc.***	
**Known GAGs** Reitsma et al. (2007), Foote et al. (2022)		**Heparan Sulfate**	Greene et al. (2003)
		**Chondroitin Sulfate**	
		**Sialic Acid**	
		**Dermatan Sulfate**	
		**Hyaluronan, Keratan Sulfate, *etc.***	

Shear mechanotransduction		
**Flow Across the Glycocalyx**	Hydrodynamic forces due to blood flow act on apical core proteins inducing a bending moment that transduces fluid shear stress Stern (2008). Tension in GAGs induced by hydrodynamic drag transmits fluid shear stress through the core proteins into the cytoskeleton Schröter et al. (1999)	Apical shear transmitted through the cytoskeleton increases tension in basal syndecan-4 proteins instigating signal transduction pathways Longley et al. (1999), Greene et al. (2003). Simulations of interstitial flow suggest magnitudes surpass necessary levels to deform the basal glycocalyx however primary evidence has not been reported Tarbell and Shi (2013)
**Cytoskeletal Remodeling**	Instigation of polymerization and polarization of actin filaments forming peripheral actin bands and stress fibers Li and Wang (2018), Thi et al. (2004)	Instigation of polymerization of actin filaments. Bayens et al. suggest syndecan-4 is critical for cytoskeletal polarization to the flow vector Longley et al. (1999), Saoncella et al. (1999), Baeyens et al. (2014)
**Flow Instigated Signal Transduction Pathways**	eNOS, CD59, SOD, HO-1, NQO1, PRX, MKP-1, Ang-2, ICAM-1, E-selectin, SREBP, TLR-2, ect. Davies (1995), Tarbell and Pahakis (2006)	Yap/Taz and Rho Elfenbein and Simons (2013), Chronopoulos et al. (2020)

## Mechanotransduction

Shear forces on endothelial cells have been extensively investigated. However, most research has been primarily focused on the apical glycocalyx as it is directly exposed to shear forces (Pahakis et al., 2007; Ebong et al., 2014; Jiang et al., 2019). Available evidence shows that even though the basal glycocalyx is not directly exposed to shear it does play a role in transducing shear forces to biochemical signals (Elfenbein and Simons, 2013; Ebong et al., 2014; Chronopoulos et al., 2020). This evidence comes from an understanding of how syndecan-4 and its heparan sulfate chains interact with basal extracellular matrix and the cytoskeleton to transduce shear forces (Chronopoulos et al., 2020; Jiang et al., 2020). Here we will primarily discuss our knowledge of syndecan-4 as little to no evidence of other proteoglycans and glycoproteins and their function in the basal cell membrane have been presented in literature. For a deeper understanding of how the endothelial cell wholistically senses and responds to shear, the basal glycocalyx must be characterized in terms of its core protein components and attached chains prior to investigation of specific transduction mechanisms.

Syndecan-4 possesses conformational structures in its cytoplasmic domain that are sensitive to tension (Elfenbein and Simons, 2013; Jiang et al., 2020). When syndecan-4 and its heparan sulfate chains bind to the extracellular matrix, tension is generated in syndecan-4, eliciting a conformational change in its cytoplasmic domain (Elfenbein et al., 2009; Chronopoulos et al., 2020). The cytoplasmic segment within syndecan-4 holds specific Rho conformational patterns, enabling its interaction with Rho inhibitors and GTPases. This unique feature allows syndecan-4 to directly influence the Rho pathway as a modulator (Elfenbein and Simons, 2013; Chronopoulos et al., 2020). With heightened tension on syndecan-4 triggered by binding with the extracellular matrix or shear due to interstitial flow, the Rho structure actives (Tada and Tarbell, 2002; Elfenbein and Simons, 2013; Tarbell and Shi, 2013; Chronopoulos et al., 2020). Other mechanical stimuli such as changes in substrate stiffness and cyclical stretch can also instigate activation of the Rho pathway (Kaunas et al., 2005; Nikmanesh, 2016; Nikmanesh et al., 2019; Chronopoulos et al., 2020). Activation of Rho prompts the liberation of Rho inhibitors, facilitating engagements with active Rho GTPases (Greene et al., 2003; Elfenbein and Simons, 2013). GTPases like RhoG, Rac1, and RhoA are in their active state when linked to enzymes such as protein kinase C alpha (PKCα). This linkage subsequently modifies the conformation of Rho in membrane structures through phosphorylation, thereby triggering additional liberation and binding of GTPases (Greene et al., 2003; Tkachenko et al., 2006; Elfenbein et al., 2009). The binding of GTPases allows for α-actin binding to syndecan-4 and stimulates actin polymerization to couple the cytoskeleton and syndecan-4 (Greene et al., 2003; Kaunas et al., 2005; Tkachenko et al., 2006; Elfenbein et al., 2009). This results in the force transmission network seen as the resting or static structure of many mechanotransduction models such as the “bumper-car” model. In this model, the apical and basal glycocalyx are connected through direct connection to stress fibers and the cytoskeleton of the endothelial cell (Li and Wang, 2018; Thi et al., 2004). However, these models are only informed by the behavior and known function of syndecan-4 and heparan chains in the endothelial cell basement membrane. Knowledge of how other GAG chains bind to syndecan-4 and other potential basal proteoglycans may implicate other known or unknown signaling pathways in the basal signaling response to shear. Exploring the impact of forces on the basement membrane, such as shear from interstitial flow and cyclic stretch, could reveal alternative signaling pathways and responses. The significant magnitudes of these forces may initiate well-established signaling pathways (Tada and Tarbell, 2001; 2002; Nikmanesh, 2016; Nikmanesh et al., 2019). The discovery of new pathways and responses in endothelial force transduction, when identified, will enhance our understanding of *in vivo* endothelial cell behavior. Additionally, this will facilitate improved *in vitro* modeling.

Syndecan-4 does not just activate the Rho pathway, it also regulates yes-associated protein (YAP) activity along with other transmembrane proteins (Baeyens et al., 2014). YAP is a transcription coactivator that has a role in converting biophysical inputs into gene expression signatures (Foote et al., 2022). In 2020 Chronopoulos et al. found that syndecan-4 mediated tension is essential for YAP transcriptional signaling at the cell extracellular matrix interface (Chronopoulos et al., 2020; Foote et al., 2022). As tension increases in syndecan-4 the RhoA pathway instigates the formation of stress fibers throughout the cell (Figure 1) transmitting tension to the nucleus simulating the movement of YAP and TAZ to the nucleus, resulting in the activation of the YAP signaling pathway (Saoncella et al., 1999; Civelekoglu-Scholey et al., 2005; Chronopoulos et al., 2020). This demonstrates that Rho activation and YAP signaling are both triggered by a syndecan-4 and integrin β1 signal disturbance (Chronopoulos et al., 2020; Foote et al., 2022).

Interactions between the Rho and YAP signaling pathways are not only instigated by syndecan-4 tension. Caveolin-1 is known to directly connect to the actin cytoskeleton in the basal section of endothelial cells and respond or activate upon mechanical stimuli (Sotodosos-Alonso et al., 2023). It is hypothesized that caveolin-1 can hold a variety of signaling molecules when in an inactive state and release them once activated with implications in YAP, Rho, and endothelial nitric oxide synthase (eNOS) signaling pathways (Frank and Lisanti, 2006; Bass et al., 2011; Sotodosos-Alonso et al., 2023). Reports indicate that basal caveolin-1 plays a pivotal role in modulating YAP and eNOS activity as well as phosphorylation within the basal cell membrane (Moreno-Vicente et al., 2018; Sotodosos-Alonso et al., 2023). Its prominence arises from its involvement in sensing substrate stiffness and initiating actin remodeling, a subject further explored in other investigations (Frank and Lisanti, 2006; Moreno-Vicente et al., 2018). However, while the role of caveolin-1 is prevalent in substrate sensing we must further explore how apical shear affects basal caveolin-1. Not only through biochemical signaling pathways but also through direct force transduction to stress fibers, which has implications for other signaling responses.

Understanding the role of the basal glycocalyx in mechanotransduction is challenging due to the limited knowledge of its composition regarding proteoglycans, glycoproteins, and GAGs. Complete characterization of the basal glycocalyx is necessary to identify the structures and GAG chains present as well as their binding patterns. Before delving deeper into mechanotransduction mechanisms, it is imperative to thoroughly characterize transmembrane proteins at the basal level. These proteins might initiate distinct signaling pathways compared to their apical counterparts or even engage in pathways that remain undiscovered to us at present.

## Remodeling

Currently, our comprehension of basal glycocalyx remodeling in response to shear stress is predominantly focused on the involvement of syndecan-4 in endothelial cell remodeling. As previously discussed, tension generated in syndecan-4 due to apical shear promotes actin polymerization altering the cytoskeletal structure (Civelekoglu-Scholey et al., 2005; Zeng and Tarbell, 2014; Chronopoulos et al., 2020). Actin polymerization results in a whole-cell remodeling response through the formation of an actin cytoskeletal network and the formation and relocation of focal adhesions (Saoncella et al., 1999; Civelekoglu-Scholey et al., 2005; Kaunas et al., 2005). Remodeling of the cytoskeleton and focal adhesions results in whole cell and tissue morphological alterations by enabling a variety of endothelial cell behaviors such as elongation, alignment, polarity, migration, and proliferation (Baeyens et al., 2014; Hu et al., 2020; Longley et al., 1999; Saoncella et al., 1999; Tkachenko et al., 2006; Zeng and Tarbell, 2014). When syndecan-4 was deleted from hypercholesterolemic mice, endothelial cells were no longer able to properly align to the direction of flow, demonstrating the role of syndecan-4 in sensing and provoking cellular reorientation along the flow vector (Baeyens et al., 2014). This is likely due to the spatial distribution of syndecan-4 and its colocalization with focal adhesions, instigating regional activation of Rho GTPases (Pankov et al., 2005; Bass et al., 2007). For example, GTPase Rac1 has been specifically noted to contribute to the control of cell polarity, alignment, and migration (Elfenbein and Simons, 2013). This process involves syndecan-4 triggering localized actin remodeling at the cell’s focal adhesion sites, leading to spatially targeted changes (Elfenbein and Simons, 2013). The altered morphological response to shear suggests that the mechanisms that instigate elongation and directional alignment are mediated by different mechanotransduction mechanisms through different proteoglycans. If the basal glycocalyx is primarily composed of syndecan-4 this suggests that the apical and basal glycocalyx components may play differing roles in sensing the magnitude and direction of shear force (Baeyens et al., 2014). Exploring additional basal proteoglycans and glycoproteins could unveil their potential involvement in initiating or mediating the morphological changes seen during the onset of shear.

**FIGURE 2:**
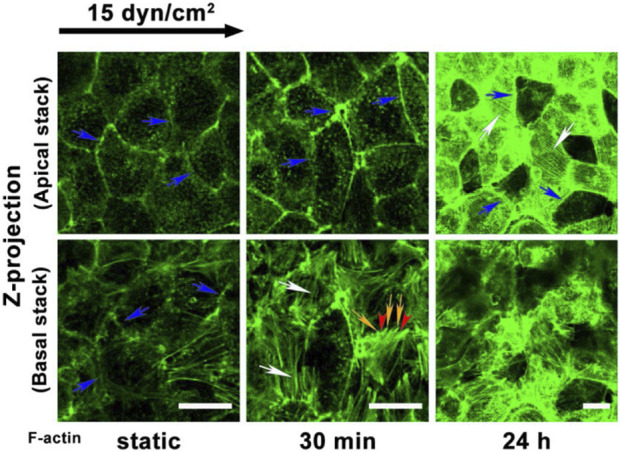
Adapted from Zeng et al., 2014 Figure 10 panel A (Zeng and Tarbell, 2014). F-actin redistribution after exposure to shear in the apical and basal where Zeng and colleagues note that blue arrows indicate the dense peripheral actin bands; white arrows indicate the stress fibers; and yellow arrows and red arrowheads denote the filopodia and lamellipodia, respectively. The apical and basal difference in cytoskeletal organization can be distinctly noted at 24 h where basal F-actin is disorganized and the apical presented long organized fibers.

The remodeling response of the cytoskeleton alone has been investigated in response to shear. Zeng et al. investigated cytoskeleton remodeling in response to shear by visualizing and quantifying F-actin distribution and organization as seen in Figure 2. After 30 min of shear exposure, new and redistributed actin microfilaments clustered at the basal and apical sides of the cell (Zeng and Tarbell, 2014). However, at 24 h the apical cell displayed many stress fibers colocalized at the surface with migratory and newly synthesized GAGs (Figure 1) (Zeng and Tarbell, 2014). The basal cytoskeleton in contrast, displayed an increase in scattered and disorganized actin microfilaments (Figure 1) (Zeng and Tarbell, 2014). The documented alterations in the apical cell have been extensively detailed and substantiated by literature (Barbee et al., 1993; Zeng and Tarbell, 2014). The colocalization of stress fibers and GAGs have been elucidated by the role of the actin cytoskeleton in supporting the synthesis of newly formed syndecan-1. However, the disorganization of filaments in the basal layer is not explained in the literature (Civelekoglu-Scholey et al., 2005; Dabagh et al., 2014; Le et al., 2021). The investigation of basal proteoglycans, glycoproteins, and the endothelial cell cytoskeleton could offer insights into the interactions between basal glycocalyx components and the cytoskeleton. Specifically, observing their colocalization at both apical and basal membranes during endothelial cells’ response to shear stress might shed light on this interaction. Presently, existing literature highlights syndecan-4 and focal adhesion structures in this context. Visualizing, localizing, and comparing basal proteoglycans and glycoproteins to the cytoskeleton, akin to the studies conducted on syndecan-1 and other proteins in the apical glycocalyx, would fulfill a gap in our understanding of the basal glycocalyx (Saoncella et al., 1999; Zeng and Tarbell, 2014).

The topological reorganization of glycocalyx components is directly tied to the organization of the cytoskeleton through their connection to stress fibers. Therefore, connecting changes in the surface organization of proteoglycans, glycoproteins, and their attached chains to cytoskeleton adaptations and the morphological response of the cell has been of interest in the field. Zeng and others’ 2013 and 2014 publications investigate the space and time-dependent reorganization and synthesis of surface glycocalyx components (Zeng et al., 2013; Zeng and Tarbell, 2014). Glycocalyx components from fat pad endothelial cells were characterized in static culture, after 30 min and 24 h of exposure to shear stress (Zeng and Tarbell, 2014). After 24 h of exposure, all measured components returned to or surpassed the levels observed in static culture (Zeng and Tarbell, 2014). The distribution of heparan sulfate and glypican-1 was comparable to the static distribution. Caveolin-1 not only displayed apical redistribution but basal as well with significant differences between the basal and apical coverage and mean florescence intensity (MFI) (Zeng and Tarbell, 2014; Harding et al., 2018). The literature extensively elucidates the dynamic reorganization and synthesis of heparan sulfate, chondroitin sulfate, glypican-1, and syndecan-1 at the apical level (Zeng and Tarbell, 2014). Particularly noteworthy is the replication and detailed explanation of the migration of heparan sulfate and the associated mobile proteoglycans to the cell boundary under flow conditions, as extensively documented (Thi et al., 2004; Koo et al., 2013; Zeng et al., 2013; Zeng and Tarbell, 2014). The movement of caveolin-1 and its synthesis in the apical cell explain the observed apical caveolin-1 dynamics, facilitating the formation of new caveolae (Figure 1). However, the literature lacks substantial support or explanation for the basal clustering of caveolin-1, highlighting yet another gap in our understanding of the basal endothelial cell function (Zeng and Tarbell, 2014).

In a follow up study to their previously discussed 2014 publication, Zeng and others examined the expression of heparan sulfate after exposure to varying levels of shear. Here syndecan-4 was explicitly investigated, and it was found that syndecan-4 expression peaked 30 min after exposure to 10 and 15 dyn/cm2 shear stress corresponding with the timeline of cytoskeletal remodeling observed in the 2014 publication (Zeng and Tarbell, 2014; Liu et al., 2016). The relationship hints that visualizing syndecan-4 alongside the cytoskeleton in the basal cell might reveal an augmented colocalized expression of syndecan-4 and F-actin following brief exposure to shear stress. However, to the best of our knowledge, a study of this nature examining this specific aspect has not yet been conducted. Hence, it is imperative to reproduce extensive research like that of Zeng and others from 2014, with a heightened focus on the fundamental elements of the basal glycocalyx. This deeper exploration is crucial for a complete comprehension of how endothelial cells respond to shear remodeling, given the basal glycocalyx’s significant role as a key influencer in intracellular remodeling and morphological alterations (Longley et al., 1999; Baeyens et al., 2014).

Integrins share many functional similarities to proteoglycans and glycoproteins as they respond to extracellular stimuli and play a significant role in cell signaling pathways (Finney et al., 2017; Chronopoulos et al., 2020; Driscoll et al., 2020). Integrins, their response to shear, and interactions with the cytoskeleton have been studied (Baeyens et al., 2014; Finney et al., 2017; Driscoll et al., 2020). This has led to many well-defined models describing how integrins mechanically couple intracellular cytoskeletal components and the extracellular microenvironment (Civelekoglu-Scholey et al., 2005; Finney et al., 2017; Driscoll et al., 2020). While it is established that elements of the basal glycocalyx are mechanically linked to the cytoskeleton and play crucial roles in signaling pathways—particularly evident in studies on syndecan-4—their interaction with the cytoskeleton has not received the same level of research emphasis as integrins (Thi et al., 2004; Le et al., 2021). This discrepancy highlights a gap in our understanding of how these glycocalyx elements engage with the cytoskeletal framework. It should also be noted that GAGs theoretically extend further beyond the cell membrane than integrins with heparan sulfate chains extending approximately 80 nm beyond their proteoglycan core protein compared to integrins total reach of 50 nm, and experience forces significant enough to promote cytoskeleton rearrangement (Sugar et al., 2016; Driscoll et al., 2020; Le et al., 2021). The similarities between integrins and glycocalyx elements in location, structure, and function provides another motivation to explore how the basal glycocalyx and underlying smooth muscle layer interact to initiate endothelial cell attachment, cell migration and alignment (Sugar et al., 2016; Driscoll et al., 2020; Le et al., 2021). Indeed, characterizing basal GAGs regarding their structure, function, and distribution is a prerequisite before gaining a comprehensive understanding of their interaction with the cytoskeleton and their broader functional roles.

## Degradation of the basal endothelial glycocalyx in pathologies

Gaining a deeper comprehension of the basal glycocalyx holds significant potential in enhancing our understanding of various pathologies like atherosclerosis, cancer, and sepsis. Particularly, exploring the degradation of the basal glycocalyx could offer crucial insights into the mechanisms underlying these diseases and potentially pave the way for novel therapeutic interventions or diagnostic strategies.

### Atherosclerosis

Atherosclerosis is the most commonly discussed pathology involving the glycocalyx as it precedes most cardiovascular disease (Lusis, 2000; Barquera et al., 2015; Hamrangsekachaee et al., 2023). It was originally assumed that atherosclerosis was a pathology inevitably associated with aging however studies have elucidated inflammatory pathways underlying the condition even in younger patients (Becker et al., 2015; Hu et al., 2021; Lusis, 2000). Changes in flow patterns in atheroprone sections of the vessel could have implications for glycocalyx expression in the basal cell membrane (Mensah et al., 2021). Atheroprone areas of the vessel are known to exhibit disturbed flow patterns (Mensah et al., 2020). These flow patterns play a significant role in atherosclerosis development and could alter basal glycocalyx structure and function (Civelekoglu-Scholey et al., 2005; Dabagh et al., 2014). Multidirectional flow patterns generate inflammatory shear forces that dysregulate the expression of glycocalyx components (Ghim et al., 2017; Deng et al., 2023). The imbalance between glycocalyx formation and degradation during the onset and progression of atherosclerosis alters the expression of genes critical to regulating the glycocalyx response to shear (Lusis, 2000; Hamrangsekachaee et al., 2023). This change results in an increase in vascular permeability through inflammatory mechanisms (Ghim et al., 2017; 2018; 2021). Ghim and others identified inflammatory mediators released by porcine aortic endothelial cells experiencing uniaxial flow that negate transcytosis mechanisms induced by multidirectional shear forces (Ghim et al., 2021). Others have elucidated transcytosis vesicular and paracellular transport mechanisms upregulated by multidirectional flow increasing permeability to macromolecules characteristic of low-density lipoproteins which form initial atherosclerotic plaques below the endothelium (Armstrong et al., 2015; Ghim et al., 2017; 2018; Ghaffari et al., 2018; Huang et al., 2019).

Dysregulation in glycocalyx formation and degradation results in alterations in glycocalyx thickness (Gouverneur et al., 2006; Koo et al., 2013; Deng et al., 2023). The balance between glycocalyx thickness and wall shear stress has been hypothesized to regulate atherosclerotic low-density lipoprotein build up in the glycocalyx (Deng et al., 2023). Increases in glycocalyx thickness and shear rate are initially atheroprotective before becoming pro-atherosclerotic when pathological levels are reached (Deng et al., 2023). Once permeability has increased the locally deposited lipoproteins begin to adhere to degraded regions of the vasculature triggering inflammatory pathways, leading to the expression of proinflammatory molecules (Becker et al., 2015). These proinflammatory molecules instigate further vessel degradation and plaque formation on the vessel wall (Becker et al., 2015). While there has not been a direct examination of the impacts of these mechanisms on the basal glycocalyx, existing evidence implies a comprehensive cellular inflammatory response. This suggests potential effects on the structure and function of the basal glycocalyx, potentially influencing transcytosis mechanisms. Indeed, while the specific involvement of the basal glycocalyx in atherosclerosis development remains unclear, Baeyens et al. have highlighted the potential significance of the basal glycocalyx in sensing flow direction, as previously discussed. Ghims’ evidence proposing multidirectional shear stress as the primary mediator of inflammatory pathways, leading to the degradation of glycocalyx and increased permeability, indicate a plausible connection between basal glycocalyx function and aspects of vascular physiology. This linkage could have implications for atherosclerosis development, particularly through syndecan-4.

Reports suggest that there is a substantial increase in syndecan-4 expression following prolonged exposure to low shear stress, a condition often associated with atherosclerosis development (Liu et al., 2016). However, exploration of syndecan-4 responses under disturbed flow patterns remains unexplored, presenting an opportunity to understand how this protein behaves under diverse flow conditions and potentially contributes to the initial development of atherosclerotic plaques. Furthermore, the effects of interstitial flow patterns on the basal glycocalyx and possible downstream implications for permeability mechanisms should be probed as a possible instigator of inflammatory mechanisms. Additionally, the impact of enzymatic and inflammatory degradation mechanisms, prevalent in atherosclerosis, on the basal glycocalyx remains unclear—a critical area requiring investigation to comprehend its potential vulnerabilities and responses in disease states.

### Cancer

Glycocalyx impairment in patients with cancer occurs due to the underlying inflammation that cancer causes (Qu et al., 2021). When blood vessels are in an inflammatory state, the glycocalyx sheds as a response (Jin et al., 2021). The inflammatory mechanisms/sheddases responsible for the shedding and degradation of the glycocalyx include Matrix metalloproteinases, Heparanase, and Hyaluronidase (Qazi et al., 2013; Krüger-Genge et al., 2019; Uchimido et al., 2019). These shedasses are activated by reactive oxygen species and pro-inflammatory cytokines such as Tumor Necrosis Factor Alpha (Wu and Zhou, 2010). The inflammatory mechasnism/sheddases degrade the glycocalx and Tumor Necrosis Factor Alpha upregulates CD44 expression (Mikami et al., 2015; Cabral-Pacheco et al., 2020). Once the GAG chains are no longer covering the CD44 receptors it easier for cancer cells to attach and complete trans-endothelial migration since there is no longer a negative charge repelling them and the barrier protection of the endothelium is lost (Qu et al., 2021; Zeng et al., 2022). Although it is understood how cancer cells migrate through the apical glycocalyx, investigating the impact of inflammatory cytokines on the basal glycocalyx and its permeability remains an unexplored area (Uchimido et al., 2019). Understanding how the basal endothelial glycocalyx responds in this inflammatory milieu could significantly enhance our understanding of basal endothelial cell permeability, shedding light on the mechanisms facilitating trans-endothelial migration of cancer cells (Cai et al., 2018).

### Sepsis

Sepsis is a systemic pathology with some similar mechanisms to cancer. Glycocalyx impairment in sepsis occurs due to inflammation caused by an extreme response to an infection (Chelazzi et al., 2015; Song et al., 2017). The inflammatory mechanisms/sheddases responsible for the shedding and degradation of the glycocalyx in sepsis are the same as in cancer; these also include Matrix metalloproteinases, Heparanase, and Hyaluronic Acid (Qazi et al., 2013; Krüger-Genge et al., 2019; Uchimido et al., 2019). Once the glycocalyx is degraded the blood vessel experiences hyper-permeability, thrombosis, augmented leukocyte adhesion and vasodilation (Uchimido et al., 2019). The glycocalyx components that are degraded circulate in the blood stream. Studies have shown that the increase of glycocalyx components in the blood stream is associated with organ dysfunction, severity and mortality related to sepsis (Uchimido et al., 2019). Akin to other pathologies discussed, the role of the basal glycocalyx in sepsis has not been explored. Exploring the impact of inflammation during sepsis on the basal glycocalyx may offer insights into the mechanisms triggering hyper-permeability, thrombosis, heightened leukocyte adhesion, and vasodilation in blood vessels. If the basal glycocalyx is identified as playing a role In pathological development, this investigation could unveil therapeutic targets for intervention.

## Discussion

**FIGURE 3:**
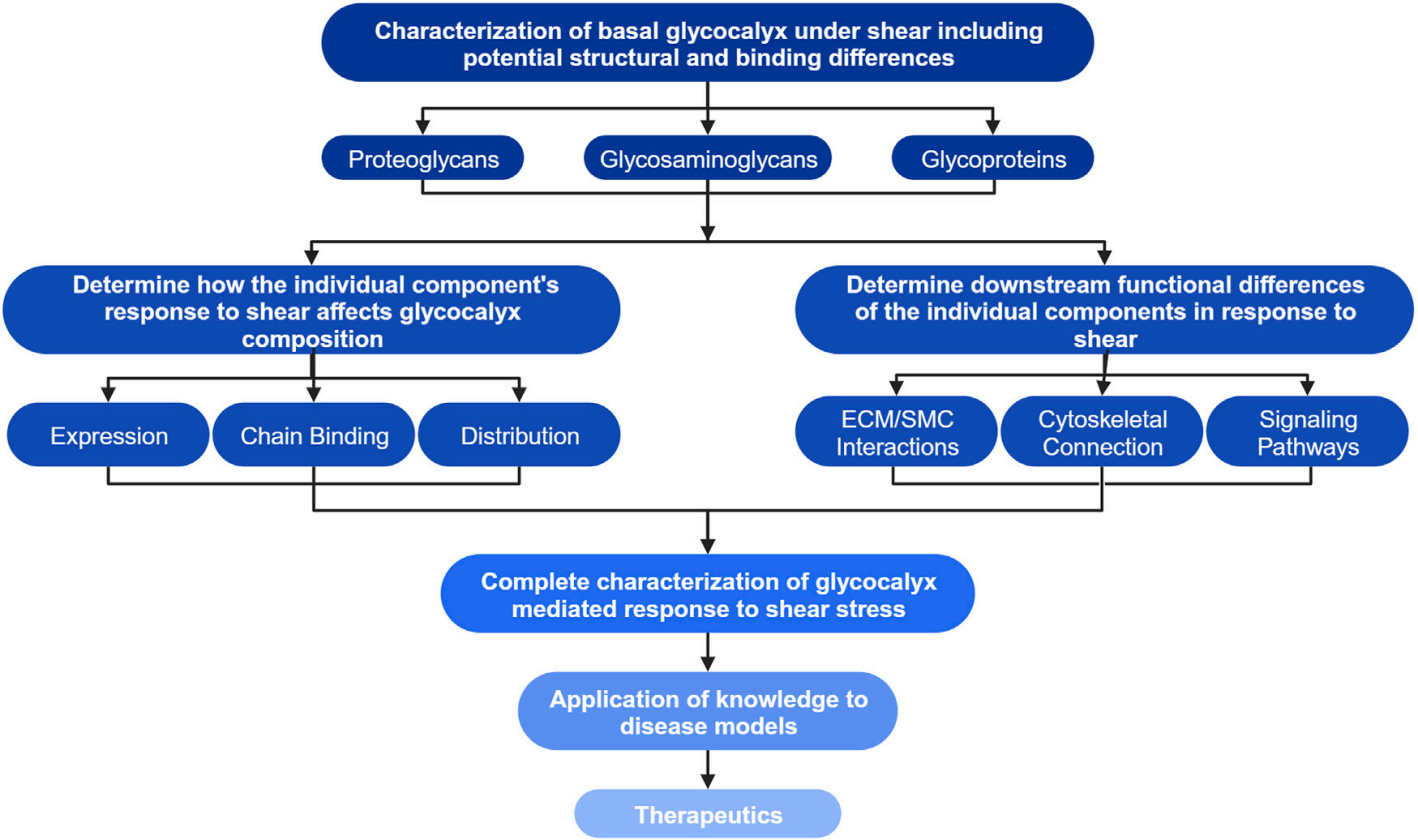
Flow diagram of research objectives to achieve a generalized model of the vascular EC’s response to shear stress. The highest level of the chart outlines the first research objectives including the characterization of basal proteoglycans, glycosaminoglycans (GAGs), glycoproteins, and any potential structural or binding differences compared to their apical counterparts. Investigation of the highest objectives may elucidate glycocalyx structures unique to the basal glycocalyx. The second layer of objectives aims to determine how individual structures characterized in the highest level of the chart affect the glycocalyx’s composition through their own alterations in expression and distribution, the secondary effects on bound glycosaminoglycan chains, as well as the downstream effects on intra- and intercellular interactions and signaling. The third level indicates that the knowledge gained through investigation of previous objectives combined with our current knowledge of the apical glycocalyx will allow for complete characterization of the glycocalyx mediated endothelial cells’ response to shear. This knowledge can then be applied to disease models and development of new therapeutics as indicated by the final levels of the flowchart. Created with BioRender.com.

Differences in the dynamic remodeling response of endothelial cells due to fluid shear stress have been noted between the apical and basal endothelial glycocalyx as shown in Figure 1 (Zeng and Tarbell, 2014; Liu et al., 2016; Zeng et al., 2018). Remodeling within the endothelial cells affects tissue organization from the local extracellular, and intracellular perspectives (Longley et al., 1999; Thi et al., 2004; Baeyens et al., 2014; Bai and Wang, 2014). However, the effects of the remodeling response of the apical and basal glycocalyx have not been independently extensively investigated, far more focus has historically been placed on the apical glycocalyx as it directly senses the fluid shear (Barbee et al., 1993; Zeng et al., 2018). New research has begun to investigate and elucidate the response and role of the basal glycocalyx in the endothelial cells’ response to shear stress (Pahakis et al., 2007; Zeng and Tarbell, 2014; Liu et al., 2016). Here the current state of literature surrounding the basal glycocalyx response to shear was discussed through the lenses of mechanotransduction and remodeling. Figure 1 depicts our current understanding of the endothelial cells’ response to shear through both the apical and basal glycocalyx. As also shown in Figure 1, there are many gaps in our current knowledge of the force transduction network that limit our understanding of the whole cell response to shear force. Before gaps such as, “How are basal proteoglycans and glycoproteins affected by apical flow?” can be adequately addressed, we must first answer more fundamental questions about basal glycocalyx composition, structure, and function in response to mechanical stimuli (Figure 3). Once these studies are conducted, it will enable the examination of structural and functional disparities within signaling pathways associated with various heparan sulfate proteoglycans, including the Rho pathway and YAP/TAZ signaling (Elfenbein et al., 2009; Bass et al., 2011; Chronopoulos et al., 2020). This deeper understanding will shed light on how different components of the basal glycocalyx contribute to and modulate these crucial signaling pathways (Figure 3). A flowchart detailing the research objectives necessary to address the major gaps in our understanding of the basal glycocalyx is presented in Figure 3. It is believed that fulfilling these research objectives will significantly enhance our knowledge of how endothelial cells respond to shear stress. This comprehensive understanding will provide insights into force transduction mechanisms and their subsequent impacts on cell morphology and behavior, ultimately advancing our knowledge in this field in the context of both health and disease.

Novel therapeutics have been developed targeting the glycocalyx in efforts to stabilize and reform the glycocalyx layer after pathological degradation. Previously, Broekhuizen et al. administered sulodexide, a compound containing both heparin and dermatan sulfate, to replace degraded glycocalyx in type 2 diabetic patients (Broekhuizen et al., 2010). They estimated the glycocalyx dimensions of two different vascular beds using sidestream dark-field imaging and combined fluorescein/indocyanine green angiography for sublingual and retina vessels and observed a significant increase in glycocalyx dimensions after treatment with sulodexide, indicating glycocalyx restoration. They also observed a decrease in plasma hyaluronidase, indicating a reduction in the shedding of hyaluronic acid from the glycocalyx (Broekhuizen et al., 2010; Masola et al., 2014). In other studies, rhamnan sulfate (a non-animal HS-like glycosaminoglycan) has been considered an HS mimetic (Maeda et al., 1991; Masola et al., 2014). Sphingosine-1 phosphate (SP1) has also been used to stabilize the glycocalyx. Zhang et al. quantified the glycocalyx in post-capillary venules of rat mesentery and measured vascular permeability in the presence and absence of S1P and found that, in the absence of S1P, the fluorescence intensity of labeled glycocalyx was 10% of that in the presence of S1P, and the permeability to albumin was 6.5-fold of that in the presence of S1P (Zhang et al., 2016). This data shows that exogenous administration of specific GCX-enhancing chemicals can either restore or strengthen the structural integrity of the endothelial glycocalyx (Zhang et al., 2016). Recently, Mensah et al. successfully replaced heparan sulfate with commercially available heparan sulfate not only structurally restoring the GAG’s presence in the apical glycocalyx but also achieving functional restoration (Mensah et al., 2017). To date, all the work done in this area has resulted in stability or restoration of the endothelial barrier function. However, the effect of these therapies on the basal glycocalyx is unknown. Should future research reveal basal glycocalyx degradation in pathologies, it could result in the development of therapeutic targets for glycocalyx-protective treatments.
