# Supplementary material for: Basal endothelial glycocalyx’s response to shear stress: a review of structure, function, and clinical implications
Source: Front Cell Dev Biol. 2024 Mar 18;12:1371769. doi: 10.3389/fcell.2024.1371769 (PMC10982814; doi:10.3389/fcell.2024.1371769)

Supplementary Material

# Supplementary Images

**Supplementary Images.** The images below contain the inputs and outputs of ChatGPT version 3.5. ChatGPT was employed to rephrase specific portions of the manuscript. These sections are distinctly marked with italicized text in the manuscript. It is essential to note that ChatGPT was utilized solely for the purpose of rewriting and enhancing clarity, without engaging in fact-checking.


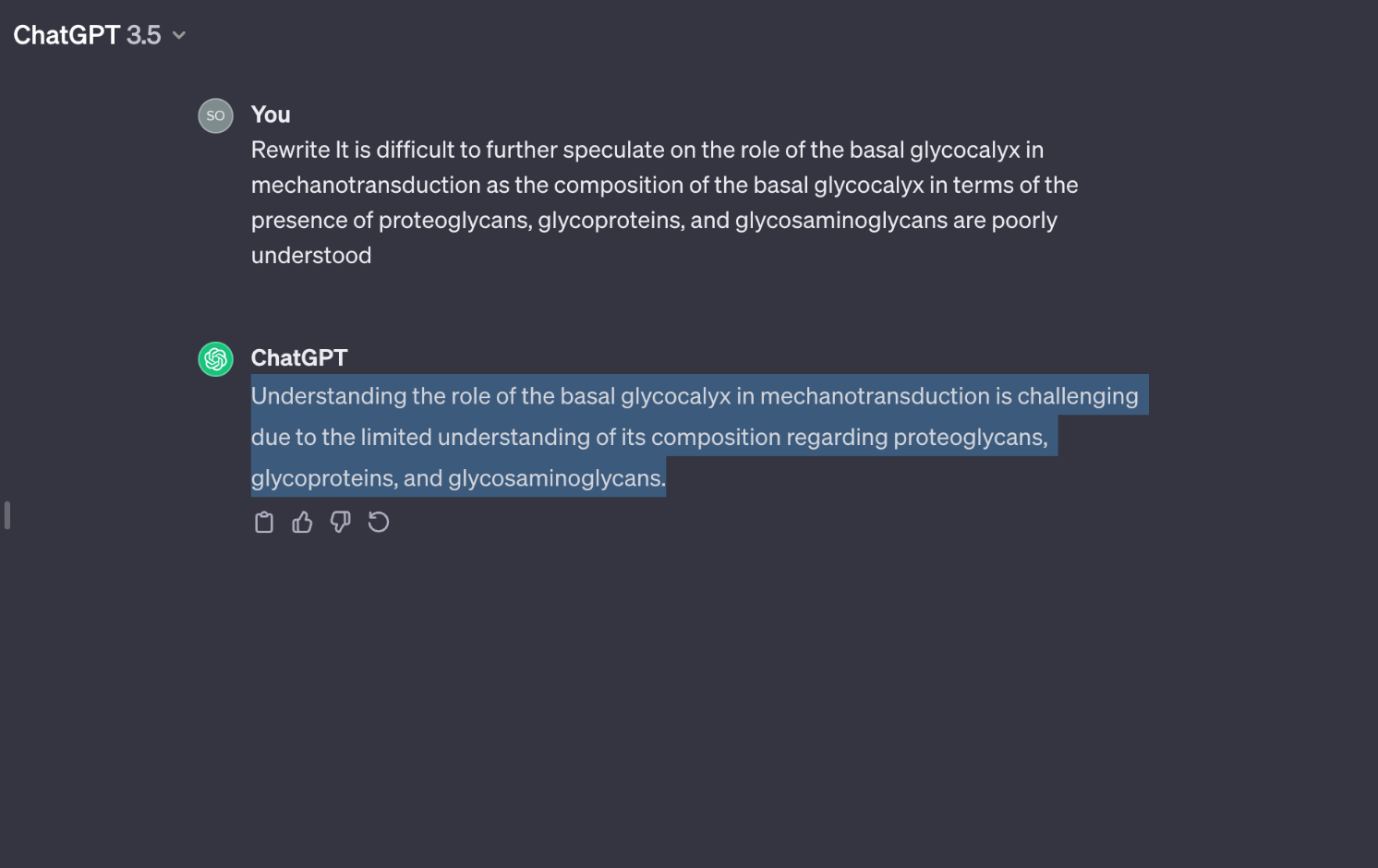


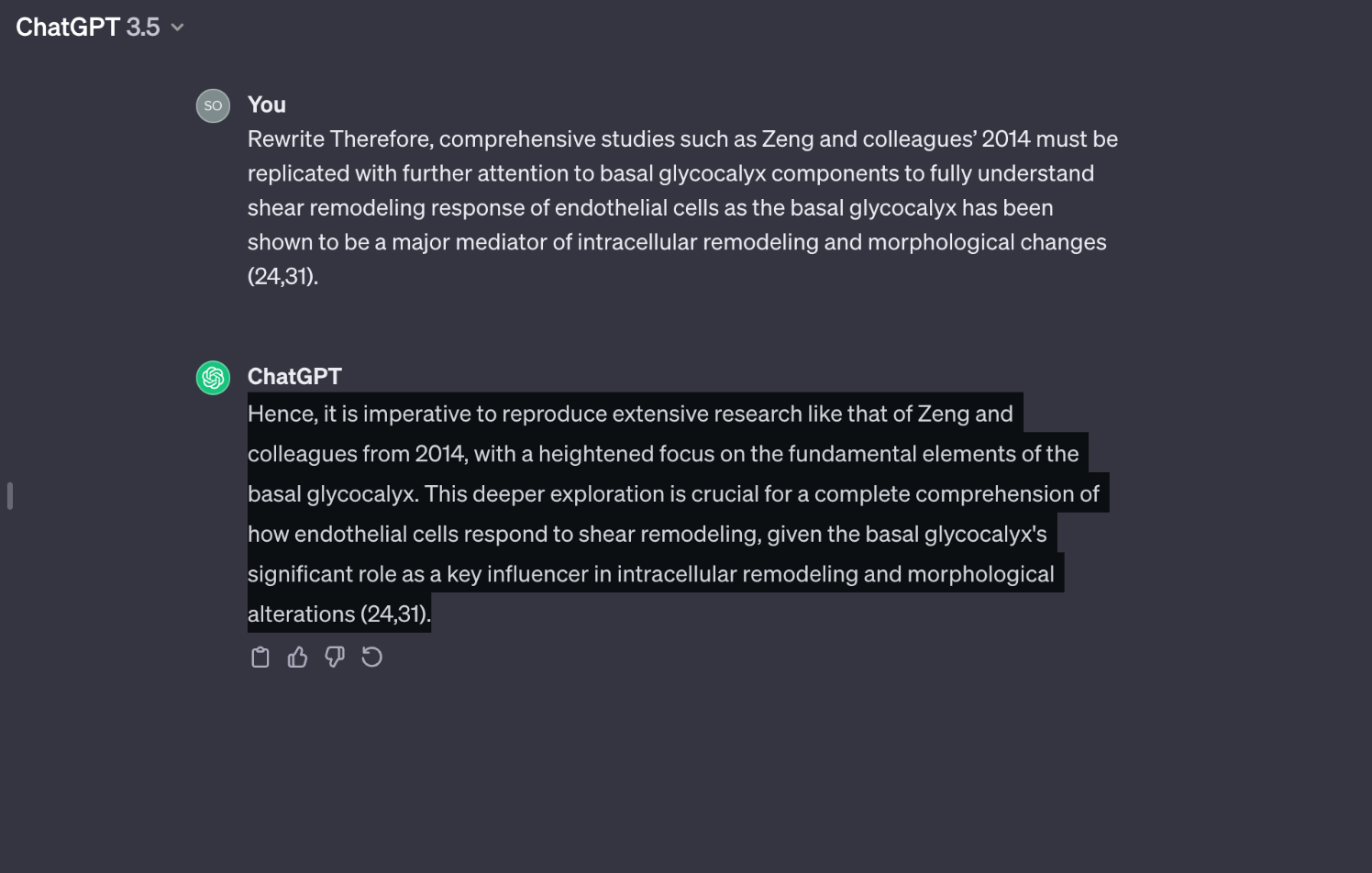

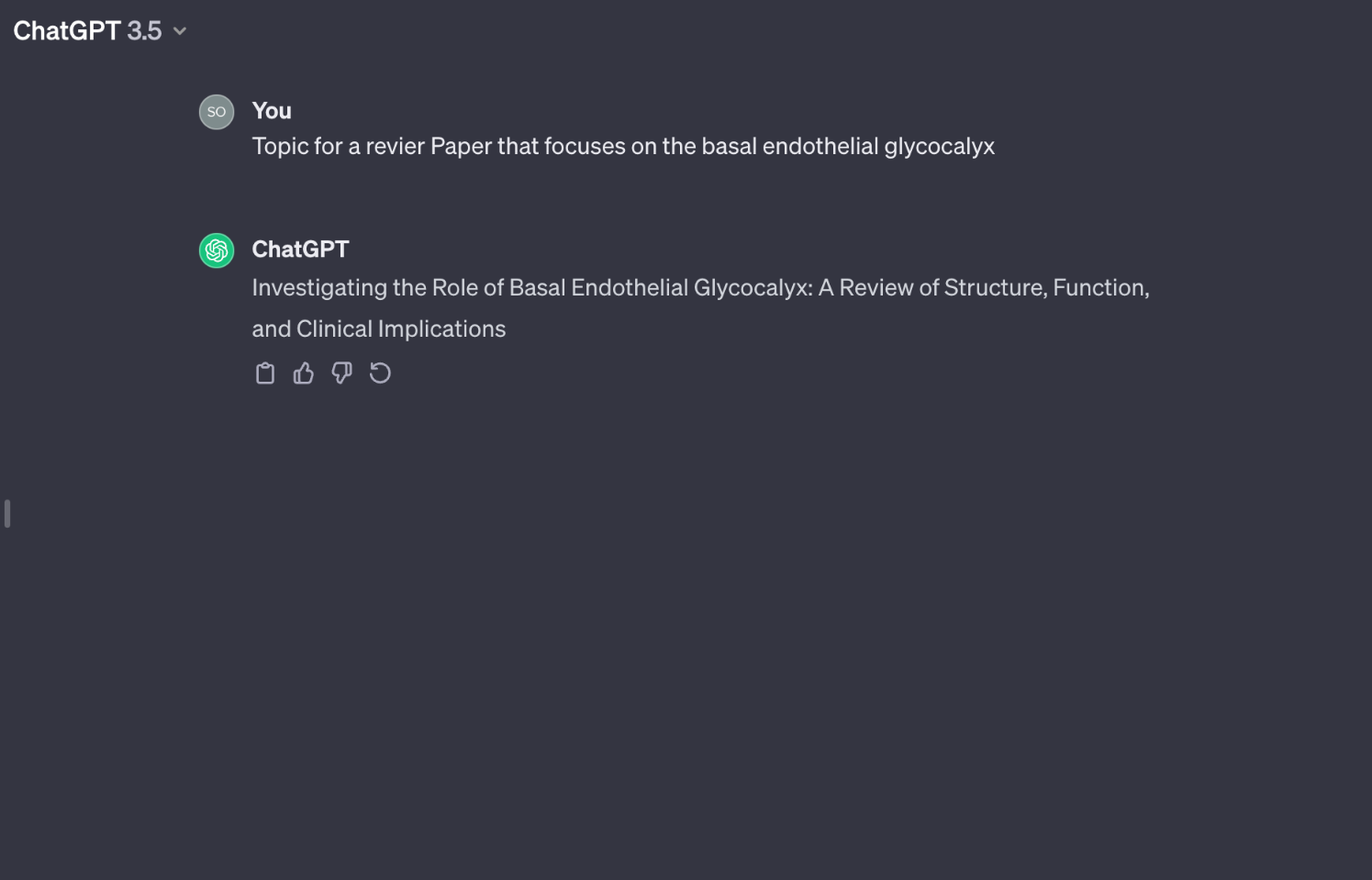

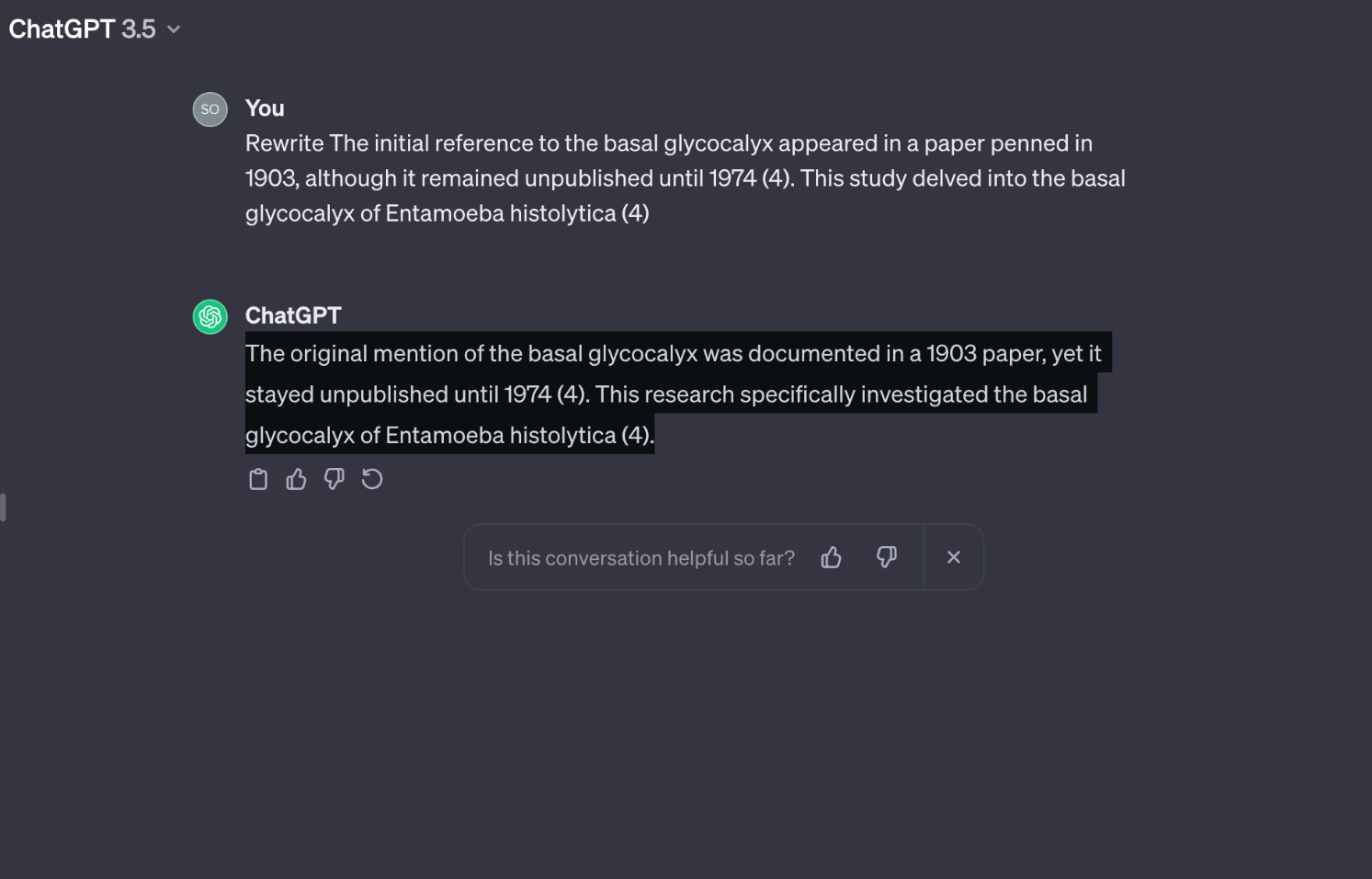

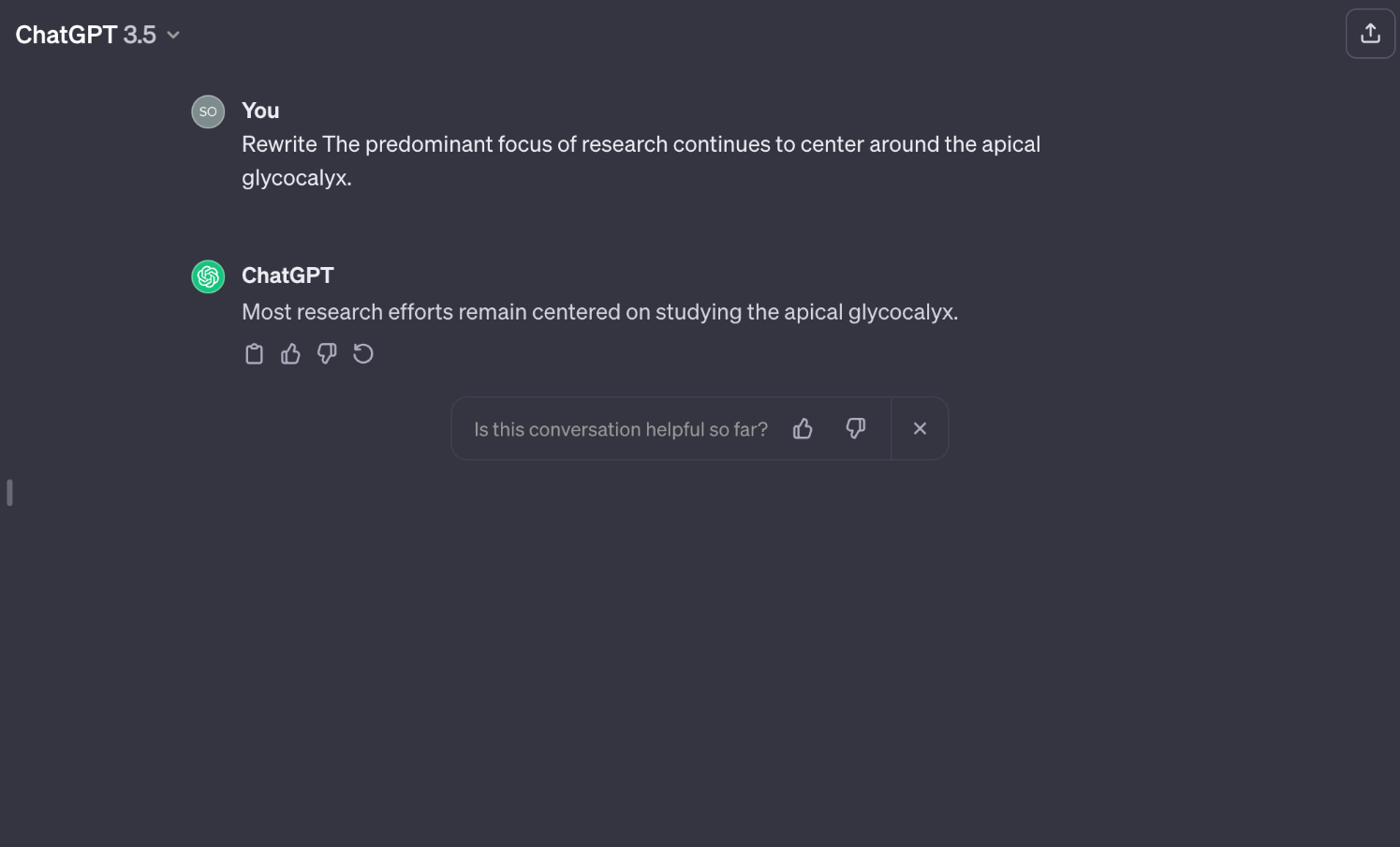

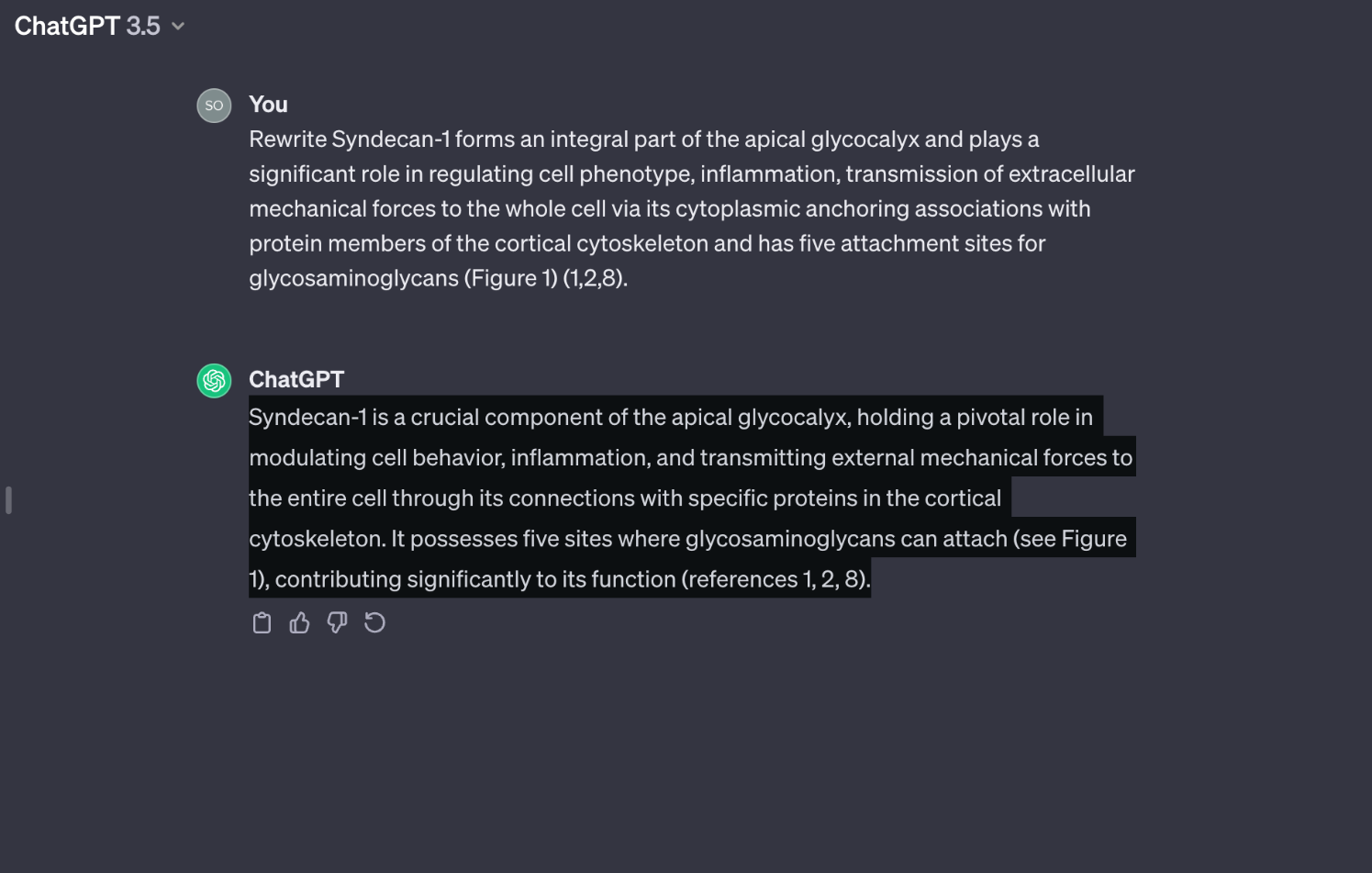

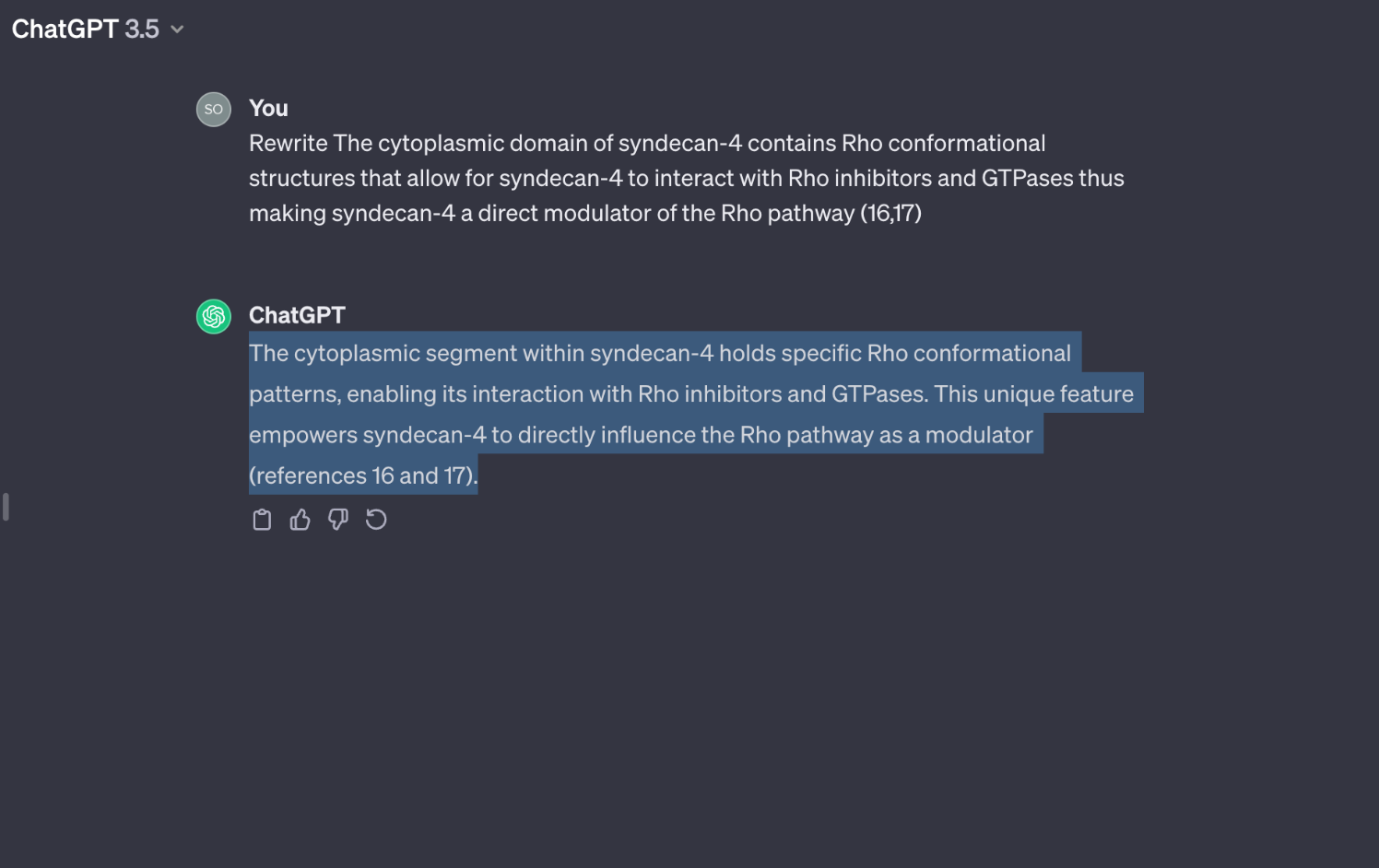

Supplement: Supplementary file 1 [file DataSheet1.docx]
